# In vitro anti-inflammatory potential and in vivo anti-arthritis activities of *Ximenia caffra* extract on antigen-induced arthritis in rats

**DOI:** 10.1038/s41598-025-32300-7

**Published:** 2026-01-06

**Authors:** Mohammed Yosri, Alsayed E. Mekky, Mahmoud M. Elaasser, Marwa M. Abdel-Aziz, Fady Sayed Youssef, Hend A. Kamel, Mahmoud M. Al-Habibi, Eman E. Helal, Basma H. Amin

**Affiliations:** 1 The Regional Center for Mycology and Biotechnology, Al-Azhar University, Nasr City, Cairo 11787 Egypt; 2 Department of Botany and Microbiology, Faculty of Science, Al-Azhar University, Cairo, 11884 Egypt; 3 Department of Pharmacology, Faculty of Veterinary Medicine, Cairo University, Giza, 12211 Egypt; 4 Microbiology and Immunology Department, Faculty of Pharmacy, Zagazig University, Zagazig, 44519 Egypt; 5 Microbiology Department, Faculty of Pharmacy and Pharmaceutical Industries, Sinai University, Kantara, Egypt; 6 Microbiology and Immunology Department, Faculty of Pharmacy (Boys), Al-Azhar University, Cairo, Egypt; 7 International Islamic Center for Population Studies and Research, Al-Azhar University, Al-Darrassa Ward, Cairo, 11651 Egypt

**Keywords:** Ximenia caffra, LC-HRMS, Antigen induced arthritis, Chondrocytes, Osteoclasts, Inflammatory cytokines, Drug discovery, Immunology, Medical research, Plant sciences

## Abstract

The current study estimated the in vitro anti-inflammatory activity and in vivo anti-arthritic activities of the aqueous ethanolic extract of *Ximenia caffra* (*X. caffra*) seeds extract. It was hypothesized that *X. caffra* seeds extract, rich in phytochemicals that could modulate inflammatory pathways and protect joint tissues in an antigen-induced arthritis rat model. The chemical composition of *X. caffra* seeds extract was examined using liquid chromatography high-resolution mass spectrometry (LC-HRMS). *Ximenia caffra* seeds extract showed promising in vitro anti-inflammatory activity with an IC_50_ = 26.01 ± 0.85 µg/ml. To evaluate in vivo efficacy, antigen-induced arthritis was established in rats using Complete Freund’s Adjuvant (CFA), followed by subcutaneous administration of *X. caffra* extract at doses of 26, 50, and 100 mg/kg body weight, alongside a standard drug control [Methotrexate (MTX), 0.3 mg/kg] in separate groups of animals. Anti-arthritic effects were assessed by measuring joint diameter, arthritic score, body weight, and through histopathological and ultrastructural analyses of joint and muscle tissues as well as osteoclast assessment, cytokine analyses, renal and kidney functions. The optimal dose (26 mg/kg) significantly alleviated arthritis symptoms, restoring joint and muscle morphology toward normal architecture. Ex vivo osteoclast evaluation and flow cytometric apoptosis analysis indicated that *X. caffra* extract promoted cellular recovery and reduced inflammatory damage. Furthermore, cytokine profiling demonstrated that treatment with *X. caffra* shifted pro-inflammatory mediators (IL-1β, IL-6, IL-17, IFN-γ) toward an anti-inflammatory balance by elevating IL-4 and regulating IgG1a/IgG2a ratios. Collectively, these results support the hypothesis that *X. caffra* seeds extract had potent anti-inflammatory and anti-arthritic effects by modulating immune responses and preserving joint integrity, suggesting its potential as a natural therapeutic alternative for rheumatoid arthritis management after further validation.

**Supplementary Information:**

The online version contains supplementary material available at 10.1038/s41598-025-32300-7.

## Introduction

Rheumatoid arthritis (RA) is a lifelong autoimmune sickness that affects about 0.2–1% of the world’s population^1,2^. It has multiple symptoms, including severe pain and joint swelling, that could develop into cartilage, joints, and bone destruction leading to disability in some cases^3–5^. RA has many systemic complications in essential body organs such as the lungs and pericardium, leading to a greater death percentage among cases with RA^6,7^. The cellular mechanisms involved in inflammatory joint diseases have advanced considerably through the use of animal models and have been alleviated in the testing of potential disease-targeting pharmaceuticals^8–10^. Various induced models of arthritis exhibit pathological features that resemble those of human disease and are widely used to investigate cellular and immunological mechanisms underlying arthritis. These induced arthritis models include: collagen-induced arthritis; antigen-induced arthritis^11^, zymosan-induced arthritis^12^, proteoglycan-induced arthritis^13^, and arthritis induced by injection of streptococcal cell wall fragments^14^. Many immune and joint-associated cells, such as chondrocytes, osteoclasts, and lymphocytes, along with pro- and anti-inflammatory cytokines, play essential roles in the pathology of RA. Chondrocytes have multifunctional roles in RA as they act as target and effector cells that upregulate the inflammatory response^15^. Osteoclasts have pivotal roles in bone resorption and the pathogenies of RA^16^. Modulating the secretion of inflammatory mediators using natural products offers an effective alternative strategy for treating RA^17^. *Ximenia caffra* is a tropical tiny tree belonging to the family Olacaceae^18^. It grows steadily in clay-rich soils and bears fruit during the winter season^19^. The plant has high protein content and contains vitamins and minerals, leading its use in many food additives. Its leaves and roots have been used as natural remedies in the cure of sterility and skin lesions as well as antimicrobial and anti-inflammatory agents^20^. The leaf extract of *X. caffra* contains many phenolic compounds, including quercetin and gallic acid. Medicinal plants, rich in phytochemicals such as terpenoids, phenolics, and flavonoids, exhibit potent anti-inflammatory and antioxidant qualities. These molecules regulate inflammatory mediators like TNF-α and IL-1β, with fewer negative effects than synthetic medicines. Long-term usage of synthetic chemicals frequently results in cardiovascular, renal, and gastrointestinal issues. Medications derived from plants can treat the symptoms and underlying causes of oxidative stress and immunological dysregulation at the same time. Owing to their low toxicity and high biocompatibility, they are suitable for long-term therapy. Furthermore, the synergistic activity of the combined compounds in plant extracts also improves the effectiveness of treatment. Their traditional usage in inflammatory disorders is supported by ethnopharmacological evidence^21^. The current study evaluates the in vitro anti-inflammatory impact and in vivo anti-arthritic activities of the aqueous ethanolic extract of *X. caffra* seeds. It was proposed that its rich content of phytochemicals of the extract could modulate inflammatory mediators and immune cell responses. Moreover, it was hypothesized that *X. caffra* seeds extract would help to restore joint integrity and reduce arthritis severity in antigen-induced arthritic rats.

## Material and methods

### Preparation of the extract

*Ximenia caffra* dry seeds were purchased from a traditional medicine shop, taxonomically identified and authenticated by an expert (Dr. Nashaat N. Mahmoud) in the Botany and Microbiology Department, Faculty of Science, Al-Azhar University (Voucher: FS00257). The seeds were cleaned and kept at room temperature until extraction. Fifty grams (50 g) of dried *X. caffra* seeds were coarsely ground (Supplementary Fig. 1) and macerated in 70% aqueous ethanol for 24 h at ambient temperature with occasional stirring. The mixture was filtered through 0.45 μm Whatman filter paper, and the filtrate was collected in a clean flask. A yield of 4.5 g of dried extract was obtained and stored at − 80 °C until further use^22^.

### LC-HRMS analysis of bioactive compounds

The separation was performed using LC-HRMS with an SB-C18 column (2.1 × 100 mm, 1.8 μm; Agilent Technologies, CA, USA). The column temperature was maintained at 25 °C. Elution was carried out using solvent A (0.1% formic acid in water) and solvent B (0.1% formic acid in acetonitrile). Gradient elution was performed at a flow rate of 200 µL/min with a 5 µL injection volume, as follows: 0–2 min, 20% B; 3–4 min, 25% B; 5–6 min, 35% B; 8–12 min, 65% B; 14–16 min, 80% B; and 20–28 min, 20% B. Mass spectrometric detection was carried out on a 3200 QTRAP mass spectrometer (AB Sciex, MA, USA) equipped with an electrospray ionization (ESI) source and a triple quadrupole–ion trap mass analyzer. Compounds were identified by comparison with the National Institute of Standards and Technology (NIST) mass spectral library (USA)^22,23^.

### In vitro anti-inflammatory testing

The aqueous ethanolic extract of *X. caffra* seeds and the reference standard Ibuprofen were tested to investigate their anti-inflammatory activity via inhibition of the lipoxygenase (LOX) enzyme from Glycine max (type I1 B). The assay was performed with slight modifications to a previously described method^24^. Briefly, in 96-well plates, 100 µL of soybean LOX solution (1000 U/mL in borate buffer, pH 9) and 200 µL of borate buffer were mixed with varying concentrations of the crude extract to obtain a final concentration range of 0.98–125 µg/mL. The reaction mixture was incubated at 25 °C for 15 min. Samples were then preincubated with 100 µL of linoleic acid (substrate) to initiate the reaction. The inhibitory activity was determined by monitoring the increase in absorbance at 234 nm using a microplate reader (BIOTEK, USA).

### Animals and treatments

Male Wister albino rats weighing 140–160 g were purchased from the animal unit of the faculty of medicine at Ain shams University, left for acclimation for two weeks, and split into six groups (nine rats each). Isoflurane (Sigma, Egypt) has been used to anesthetize rats at a level of 1–3%. The first group served as the negative control (NC) and received subcutaneous injections of saline and 10% Tween 80, respectively. The other groups were injected subcutaneously at the base of the tail with 100 µL of complete Freund’s adjuvant (CFA; Sigma-Aldrich, USA) twice weekly for 15 days to induce an arthritis model^25^. After the establishment of the antigen-induced model, signs of arthritis appeared in the remaining rat groups. The second group represented the positive control (PC) and received no treatment. The third, fourth, and fifth groups were administered *X. caffra* seeds extract at doses of 26, 50, and 100 mg/kg body weight (b.w.), respectively, via subcutaneous injection. The sixth group received the anti-rheumatic standard drug methotrexate (MTX; Orion Pharma, Espoo, Finland) at a dose of 0.3 mg/kg b.w., administered subcutaneously twice per week^26^. The *X. caffra* seeds extract was administered three times per week for two weeks. All animal experiments were approved by the Ethical Committee of the Regional Center for Mycology and Biotechnology (Approval No. RCMB22062020). All procedures were conducted in accordance with the ARRIVE guidelines and the National Institutes of Health Guide for the Care and Use of Laboratory Animals (NIH Publication No. 8023, revised 1978).

### Assessing swelling and mobility scoring

The clinical signs of inflammation were monitored daily from the first day after arthritis induction until the end of the experiment. The severity of inflammation was classified into six levels as follows: level 0 (inflammation score = 0), level 1 (inflammation score between 0.1 and 0.9), level 2 (inflammation score between 1 and 1.9), level 3 (inflammation score between 2 and 2.9), level 4 (inflammation score between 3 and 4.9) and level 5 (arthritis score of 5.0 and more than 5.0). The arthritis score was evaluated alongside joint swelling, which was measured using a digital caliper (Fischer Darex, France). Body weights were recorded regularly and expressed as mean values for all six rat groups^27,28^.

### Histopathology studies

Rats were euthanized by cervical dislocation, and the tibiofemoral joints and hind limb muscles were harvested. The tibiofemoral joint samples were decalcified using 10% ethylenediaminetetraacetic acid (EDTA; Thermo Fisher, USA) for two weeks. The tissues were then sectioned into 10 μm-thick slices and processed for histopathological examination. Slides were stained sequentially with hematoxylin and eosin (H&E; Fisher Scientific, USA), then dehydrated, mounted, and air-dried at room temperature for several days before imaging. Histological images were captured at 40× magnification using a Zeiss microscope and imaging system (Carl Zeiss Inc., Germany)^29^.

### Transmission electron microscopy

Decalcified tibiofemoral joint and hind limb muscle samples were collected to be processed for transmission electron microscopy. Samples were fixated with 2% glutaraldehyde and 1% osmium tetroxide, dehydrated with acetone, embedded with epoxy resin, sectioned using an ultra-microtome, and stained. The different cellular structures were then visualized by transmission electron microscopy (JEOL, Japan)^30^.

### Cell culture studies

Bone marrow-derived monocyte/macrophages were isolated from the tibias of animals by scrub the bone-marrow hollow with RPMI-1640 medium. The cells were brood for 6 h to split non- adherent from adherent cells. Non-adherent cells were then cultured in 48-well plates at 2 × 10^5^ cells/well and cultivated in the existence of 10 ng/mL rh M-CSF (Life Science, USA) for 3 days to macrophage-like osteoclast precursor cells. After 3 days, the non-adherent cells were washed, and pre-osteoclasts were cultivated in the presence of 10 ng/mL M-CSF, 50 ng/mL, RANKL, and various levels of sodium butyrate for 4 days to induce the formation of osteoclasts. On the second day, the medium was replaced with fresh medium contained M-CSF, RANKL, and sodium butyrate. Photos were taken using inverted microscopy (Zeiss microscope and imaging system, Carl Zeiss Inc., Germany)^31,32^.

### Flow cytometry analysis

Cultured osteoclasts were de-aggregated using trypsin in 0.25% pancreatin and washed using phosphate-buffered saline (PBS). The death rate was assessed by an Annexin V-FITC and propidium iodide (PI) staining kit (BD Bioscience, USA). The cells were suspended in a buffer has PI stock solution and kept away from light at room temperature for 15 min. The cells were examined by flow cytometry (BD Bioscience, USA)^33,34^.

### ELISA assays

The serum titers of cytokines including IFN- γ, IL-1β, IgG1a, IgG2a, IL-4, IL-6, and IL-17 were quantified by an ELISA kit (Invitrogen, Thermo Fisher Scientific, MI, USA) according to the manufacturer’s protocol.

### Biochemical assays

The levels of serum alanine aminotransferase (ALT), aspartate aminotransferase (AST), creatinine, and urea have been detected in the serum that collected from all examined animal groups using (Human Diagnostic Company, Germany) kits according to the manufacturer’s protocol^35^.

### Statistical analysis

Analyses were performed using GraphPad Prism software (Version 5). For comparisons, an unpaired Student’s *t* test and one-way analysis of variance (ANOVA) followed by Tukey’s post hoc test were used; the significance level was measured at *p* < 0.05.

## Results and discussion

### Identification of compounds in ***X. caffra*** seeds extract

The following compounds were positively identified using 198 LC-HRMS: citric acid with m/z (191, 111, 87 and 173), Gallic acid with m/z (125, 169 and 111), aconitic acid with m/z (111), procyanidin B1 with m/z (289, 407, 425 and 577), *p*-Coumaorylquinic acid with m/z (163, 119, 191 and 337), catechin with m/z (289, 125, 203, 245 and 151), procyanidin B2 with m/z (289, 407, 425 and 577), epicatechin at retention time 13.57 min., quercetin galloyl glucoside with m/z (300, 615, 463, 255 and 169), quercetin galloyl glucoside with m/z ( 300, 463 and 615), quercetin-3-O-robinobioside with m/z (300, 609, 271 and 125), rutin with m/z (300, 609, 271 and 255), hyperoside with m/z (300, 463, 271, 255), isoquercitrin with m/z (300, 463, 271,301 and 255), quercetin-3-O-glucoside with m/z (300, 271, 463, 255 and 125), kaempferol glucoside with m/z (285,169 and 447), luteolin-7-O-glucoside with m/z (285, 284, 169,125 and 447), trilobatin with m/z (315 and 345), quercetin- 3-O-pentoside with m/z ( 300, 271, 255, 433 and 315), quercetin rhamnoside with m/z (300, 271, 255 and 447), quercetin rhamnoside with m/z (300, 271, 255, 447, and 243), quercetin with m/z (125 and 169), hesperetin at retention time 24.49 min., dihydroxy hexadecenoic acid with m/z (287) (Table 1, Supplementary Fig. 2). In the present study, the aqueous ethanolic extract of *X. caffra* seeds yielded various compounds with diverse levels where, catechin, citric acid, hyperoside, isoquercetin, procyanidin B1, kaempferol glucoside, quercetin-3-O-robinobioside, and rutin were the most frequent bioactive compounds with highest concentrations in extract. The findings are in same line with other reports^22,36^ who used ethanol to extract efficient antioxidant and anti-inflammatory compounds as solvent with minimal cytotoxicity.

**Table 1:** LC-HRMS for identified compounds obtained for from *X. caffra* seeds extract.

No.	Retention time (min)	MS^E^ fragments^a^	m/z^b^	Compound name	Chemical Formula	Concentration (mg 100 ml^− 1^)
1	3.12	191,111,87,173	191.0187	Citric acid	C_6_H7O_7_	295.2
2	5.8	125,169,111	169.0129	Gallic acid	C_7_H_5_O_5_	5.56
3	9.14	111	173.0089	Aconitic acid	C_6_H_5_O_6_	N/A^c^
4	10.68	289,407,425,577	577.1344	Procyanidin	C_30_H_25_O_12_	N/A^c^
5	10.68	289,407,425,577	577.1317	Procyanidin B1	C_30_H_25_O_12_	12.2
6	11.18	163,119,191,337	337.0916	p-Coumaorylquinic acid	C_16_H_17_O_8_	N/A^c^
7	11.48	289,125,203,245,151	289.0713	Catechin	C_15_H1_3_O_6_	2.66
8	12.72	289,407,425,577	577.1345	Procyanidin B2	C_30_H_25_O_12_	0.25
9	13.57	Weak	289.0698	Epicatechin	C_15_H_13_O_6_	N/A^c^
10	16.63	300,615,463,255,169	615.0979	Quercetin galloyl glucoside	C_28_H_23_O_16_	N/A^c^
11	16.94	300,463,615	615.0977	Quercetin galloyl glucoside	C_28_H_23_O_16_	N/A^c^
12	17.06	300,609,271,125	609.1432	Quercetin-3-O-robinobioside	C_27_H_29_O_16_	0.12
13	17.27	300,609,271,255	609.1458	Rutin	C_27_H_29_O_16_	0.37
14	17.51	300,463,271,255	463.0878	Hyperoside	C_21_H_19_O_12_	0.96
15	17.51	300,463,271,301,255	463.0876	Isoquercitrin	C_21_H1_9_O_12_	1.09
16	17.81	300,271,463,255,125	463.0886	Quercetin-3-O-glucoside	C_21_H_19_O_12_	0.11
17	18.06	285,169,447	447.0938	Kaemferol glucoside	C_21_H1_9_O_11_	0.60
18	18.06	285,284,169,125,447	447.0935	Luteolin-7-O-glucoside	C_21_H_19_O_11_	0.02
19	18.75	315,345	435.1284	Trilobatin	C_21_H_23_O_10_	0.07
20	18.83	300,271,255,433,315	433.0764	Quercetin-3-O-pentoside	C_20_H1_7_O_11_	N/A^c^
21	18.89	300,271,255,447	447.0927	Quercetin rhamnoside	C_21_H1_9_O_11_	N/A^c^
22	19.63	300,271,255,447,243	447.0927	Quercetin rhamnoside	C_21_H_19_O_11_	N/A^c^
23	23.99	125,169	301.0353	Quercitin	C_15_H_9_O_7_	0.02
24	24.49	Weak	301.1643	Hesperetin	C_15_H_25_O_6_	N/A^c^
25	24.5	287	287.2236	Dihydroxy hexadecanoic acid	C_16_H_31_O_4_	N/A^c^

^a^Most common fragment stated first.

^b^The mass accuracy for all compounds was better than 5 ppm.

^c^ Concentrations of Epicatechin, Hesperetin and all identified from 17 to 25 compounds could not be determined.

### In vitro anti-inflammatory activity

**Fig. 1:**
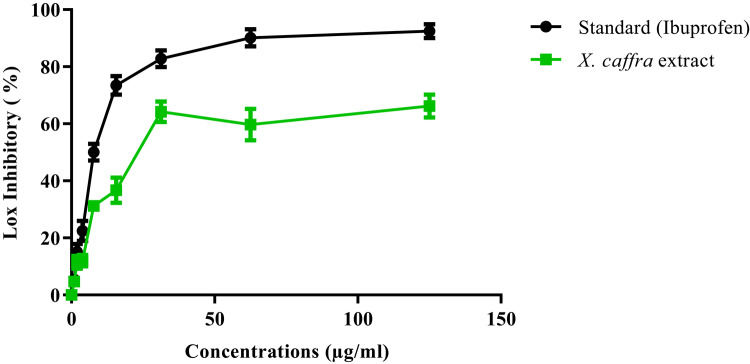
In vitro anti-inflammatory effect of a 70% aqueous ethanolic extract of *X. caffra* seeds compared with Ibuprofen standard. Values are expressed as mean ± SEM (*n* = 3).

The results indicated that the aqueous ethanolic seeds extract of *X. caffra* significantly (*p* ≤ 0.05) inhibited the LOX enzyme, with a promising inhibitory impact (IC_50_ = 26.01 ± 0.85 µg/mL) compared to Ibuprofen, as a reference compound (IC_50_ = 1.5 ± 1.3 µg/mL), respectively. In particular, *X. caffra* seeds extract possesses anti-inflammatory potential in a dose dependent manner, as shown in (Fig. 1). The aqueous ethanolic extract of *X. caffra* seeds showed a promising in vitro anti-inflammatory effect, attributed to its phenolic and flavonoids content. In accordance with Tremocoldi et al.^37^, who reported the pharmaceutical anti-inflammatory role of avocado due to its content of procyanidin B1, catechin, and epicatechin. Additionally, Chen et al.^38^ explained the action of rutin in the treatment of wound in diabetic animal model through an anti-inflammatory response. Furthermore, Shen et al.^39^ examined the role of isoquercitrin in controlling oxidative and inflammatory responses in rat muscles model. Moreover, Abdallah et al.^40^ reported the anti-inflammatory role of kaempferol-7- *O*-*β*-glucoside, cuneatannin, and 2*R*-naringenin from *Euphorbia cuneata* extract in pulmonary infected animals. Lastly, Sun et al.^41^ reported both in vitro and in vivo anti-inflammatory effects of hyperoside.

### Effect of ***X. caffra*** extract on digital scoring of disease

**Fig. 2:**
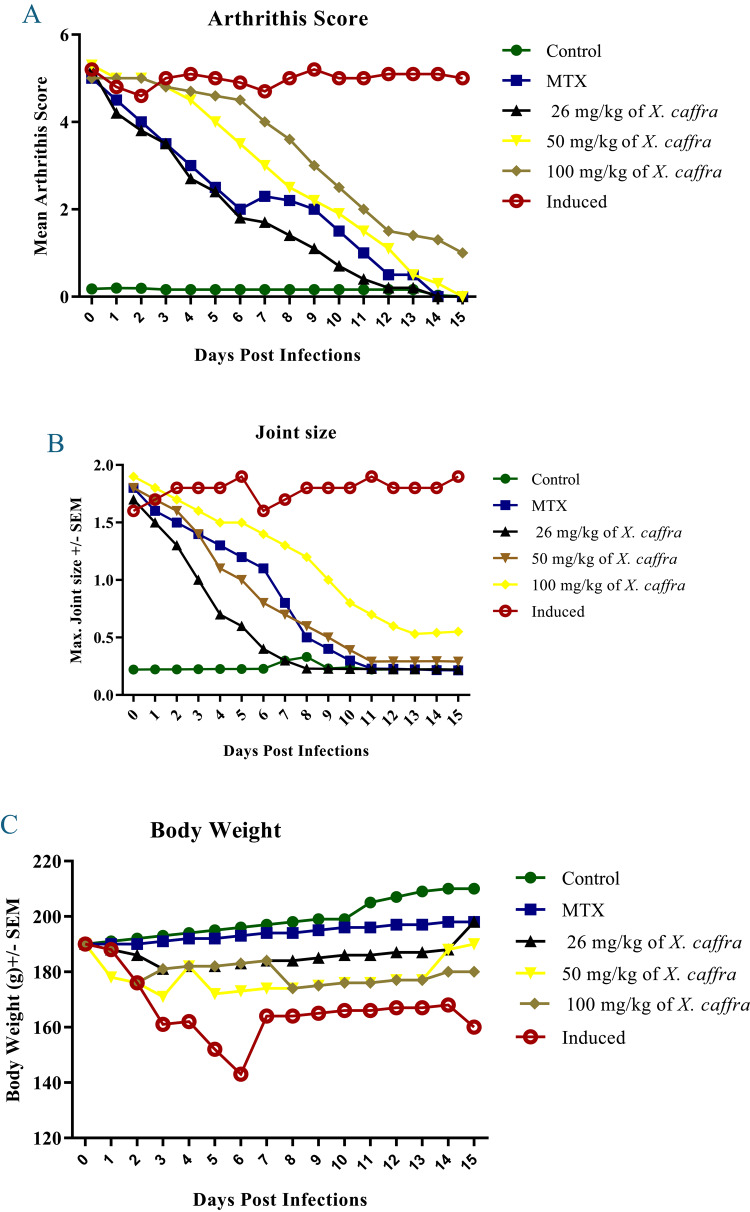
Standard methods were used for 15 days after disease induction. (**A**) Experimental arthritis scores of tested groups comparing levels upon using 26, 50 and 100 mg/kg *X. caffra* seeds extract versus negative, induced and of positive control groups. Additional conventional measurements were manually collected in the present model, such as joint measurements (**B**), and body weight (**C**) confirmed effectiveness of treatments. ∗*P* ≤ 0.05 vs. control. (*n* = 3).

Arthritis score, joint size, and body weight have been used to assess the development of disease in the present model of antigen-induced arthritis. On Day 0, animals were induced using CFA twice a week for 15 days, while the negative control group was administered saline and 10% Tween-80, showing no signs of inflammation, regular joint size, and a steady increase in body weight as the rats maintained normal feeding behavior until the end of the experiment. However, ankle joint sizes, arthritis scores, and body weight differed significantly (*P* ≤ 0.05) in induced rats which received no treatment compared to negative control rats after three days post-induction (Fig. 2A–C). Treatment with 26 and 50 mg/kg of *X. caffra* seeds extract in the separate animals’ groups significantly reduced (*P* ≤ 0.05) ankle joint size and arthritis scores, showing effects comparable to those of the MTX-treated group, and restored near-normal measurements by the end of the experiment. Administration of 100 mg/kg of *X. caffra* seeds extract had a marked effect on all tested parameters, resulting in minimal inflammation throughout the study period. Furthermore, body weight was enhanced in the tested groups, confirming the effectiveness of the treatments. This model designed to study joint structural alterations, characterized by pannus formation and infiltration of various cell types, which are early manifestations of rheumatoid arthritis^42^. This report studied the anti-inflammatory effect of different doses of *X. caffra* seeds extract in AIA rats to primarily illustrate the role of chondrocytes, osteoclasts and various inflammatory cytokines in the investigated model. Administration of *X. caffra* seeds extract produced an early reduction in inflammatory signs in a dose-dependent manner, consistent with Alamgeer et al.^43^, who reported that *Berberis calliobotrys* extracts improved inflamed paw conditions by the 15th day of treatment. Similarly, *Alternanthera bettzickiana* ethanolic extract significantly reduced arthritis symptoms compared to normal rats^44^, while Yang et al.^45^ reported restoration of normal body weight following treatment with *Pterospermum heterophyllum* extract in experimental animals.

### Histopathology and TEM examinations

**Fig. 3:**
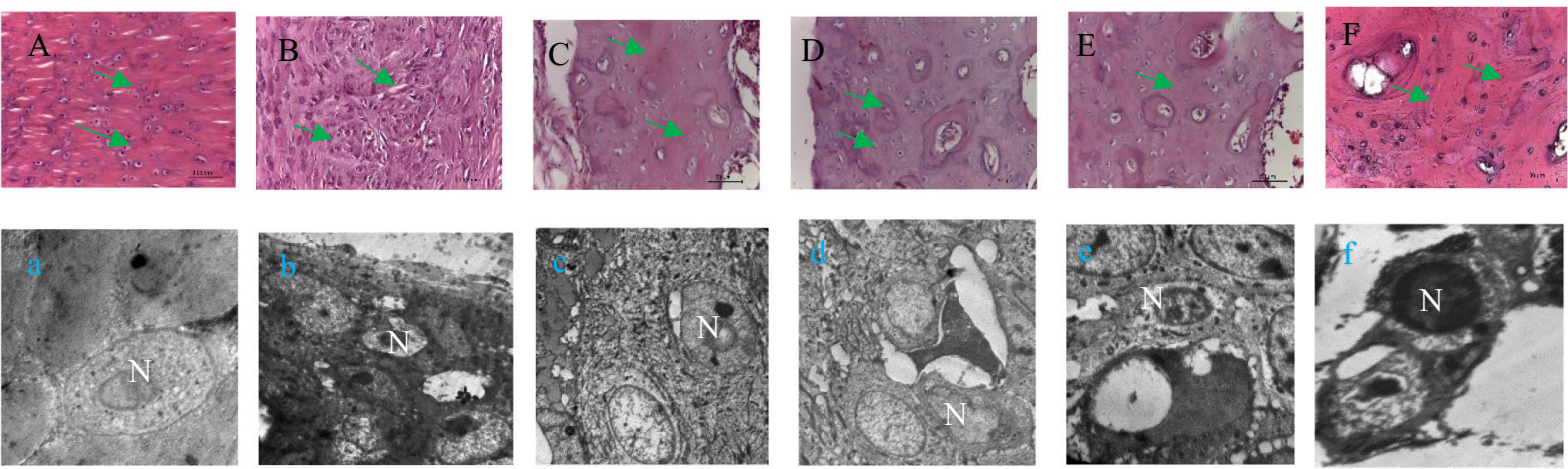
H&E staining and transmission electron micrographs of rat’s knee joints. Control (**A**, **a**), AAI (**B**, **b**) knee joint. (**F**, **f**), served as positive control where induced animals received standard drug [MTX; 0.3 mg/kg BW]. Where chondrocytes are regular shapes arranged in parallel rows (green arrows) in the normal group, while chondrocytes shrink (green arrows) in infected animals with irregular nucleus shape and pannus reaction in the induced rats. Note a decrease number of hypertrophic chondrocytes (green arrows) in the cartilage zone in positive control group as compared to the control knee. (**C**, **c**; **D**, **d**; **E**, **e**) displaying inflammatory cellular buildup and differentiated cells and nodular necrosis. Where 26, 50 and 100 mg/kg *X. caffra* seeds extracts were used respectively with induced animals and caused the tissue injury with minor inflammation (magnification 20× to images **A**–**F** & 8000× to images **a**–**f**).

Sections from the normal (negative control) group showed that the knee joint contained typical articular hyaline cartilage with elongated chondrocytes exhibiting a regular nuclear structure (Fig. 3A, a). In contrast, the antigen-induced arthritic group showed irregular and condensed synovial tissue containing numerous inflammatory cells and exudates, as well as shrunken, degenerated chondrocytes with dark, eccentric nuclei (Fig. 3B, b). Treatment with 26 mg/kg of *X. caffra* seeds extract enhanced chondrogenic perichondrium differentiation, resulting in regularly preserved chondrocytes with normal nuclei and cytoplasm, thereby restoring normal joint structure (Figs. 3C, c). Sections from rats administered 50 and 100 mg/kg of *X. caffra* seeds extract showed differentiated cells with mild nodular necrosis, irregular chondrocyte nuclei, and vacuolated cytoplasm, suggesting that these higher doses were suboptimal for proper cellular organization (Fig. 3D, d, E and e). In contrast, sections from the MTX-treated group showed a reduction in peripheral chondrocytes and slightly irregular nuclear morphology in the articular cartilage (Fig. 3F, f).

Chondrocytes are enclosed within the extracellular matrix of articular cartilage, which contains various proteins such as collagen type II and hyaluronan^46^. It has been shown that curcumin, in vitro, downregulates IL-1β and MMP-3 production, thereby enhancing the gene expression of IL-6 and IL-8 in normal chondrocytes^47^. In the present study, *X. caffra* seeds extract exhibited a regenerative effect on chondrocytes in rats’ joints, in agreement with Abdel-Azeem et al.^48^, who reported that secondary metabolites isolated from *Chaetomium globosum* helped preserve chondrocyte structure. Totoson et al.^49^ reported muscle proteolysis in the rectus femoris and tibialis anterior muscles, accompanied by inflammatory cell infiltration on day 40 after induction in rats. Consistent with our findings, an inflammatory response associated with structural alterations in muscle tissue was observed. Moreover, treatment with graded doses of *X. caffra* seeds extract reversed these harmful effects, leading to the restoration of normal movement in the animals.

**Fig. 4:**
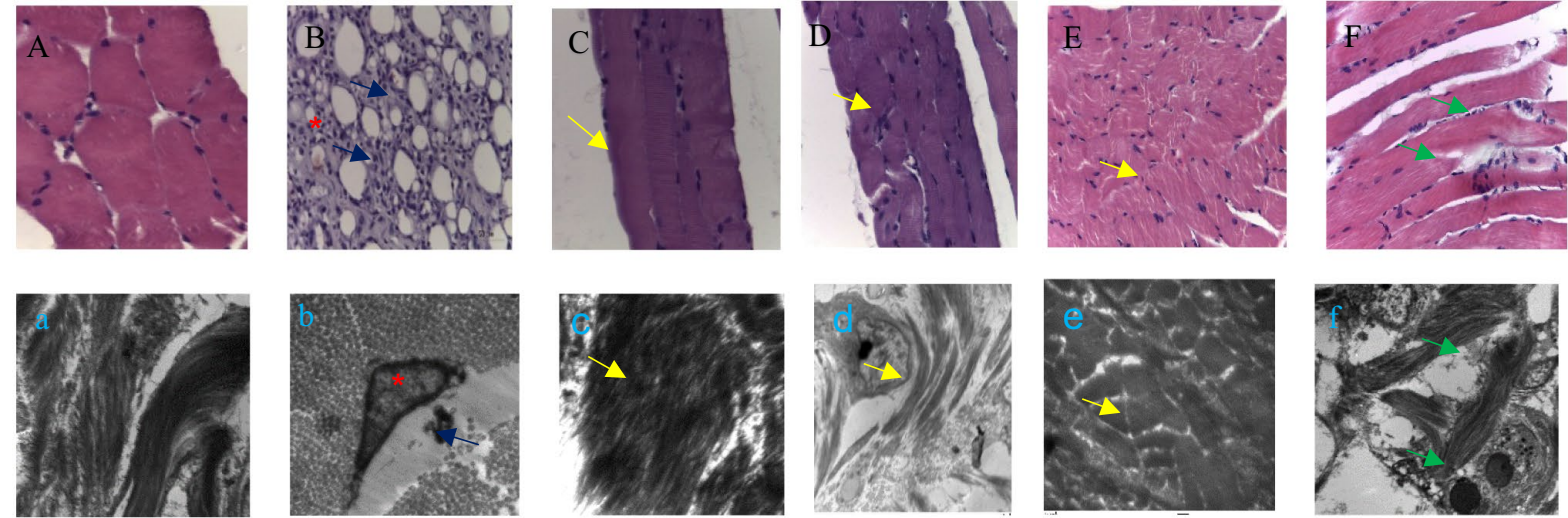
Photomicrographs of muscles taken from control rats or those submitted to adjuvant-induced arthritis (AIA). In the control groups, the muscles exhibited normal morphology (**A**, **a**). In AIA group, the muscle showed signs of damage where asterisk: nuclear aggregations; blue arrow: inflammatory infiltrate (**B**, **b**). While induced animals were treated with 26, 50 and 100 mg/kg *X. caffra* seeds extract respectively showing normal muscle fibers (yellow arrows) (**C**–**E**) & (**c**–**e**). The last group where induced animals treated with MTX as standard drug showed slight necrosis in muscles (green arrow). Where (**A**–**F**) Staining: hematoxylin and eosin (20× magnification) while (**a**–**f**) Transmission electron micrographs (8000× magnification).

To evaluate the effect of graded doses of *X. caffra* seeds extract on the morphological structure of hind limb muscles, tissue samples were collected, processed, and stained with hematoxylin and eosin, and prepared for transmission electron microscopy examination. In the negative control group, classical muscle structure was observed, characterized by regular fibers and nuclei with homogeneous distribution (Fig. 4A, a). However, in antigen-induced arthritic animals, severe inflammation, myofiber degradation, reduced muscle mass, decreased fiber size, and the presence of large vacuoles were evident (Fig. 4B, b). Treatment with 26 mg/kg of *X. caffra* seeds extract in the induced rats restored the normal organization of muscle bundles, showing numerous fibers with regularly distributed nuclei (Fig. 4C, c). Furthermore, administration of 50 and 100 mg/kg of *X. caffra* seeds extract resulted in reduced cellular infiltration, although fibers appeared irregular with numerous centrally located nuclei (Fig. 4D, d, E and e). In comparison, muscles from MTX-treated rats showed slight necrosis and mild nuclear aggregation (Fig. 4F, f).

### Effect of ***X. caffra*** extract on ex vivo cultured osteoclast

**Fig. 5:**
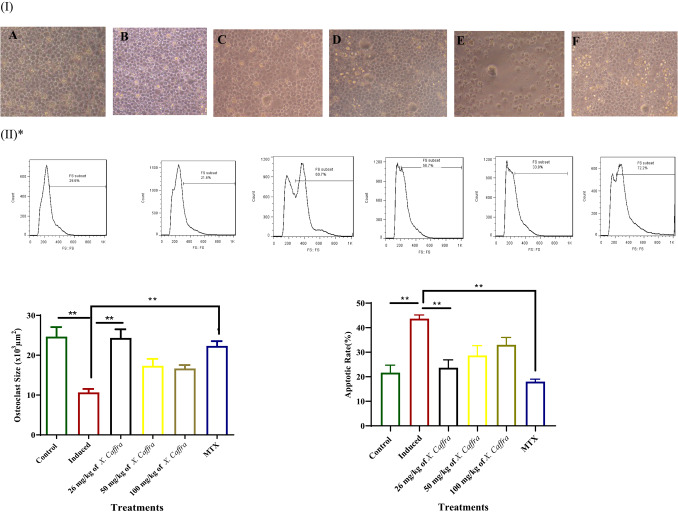
I-Microscopic examination of prepared osteoclasts from rat bone marrow progenitors (magnification 200X) where (**A**) Control Cells, (**B**) Cells cultured from induced rats. (**C**, **D**, **E**) Cells cultured from induced rats and treated with *X. caffra* seeds extract 26, 50 and 100 mg/kg respectively and (**F**) Cells cultured from induced rats and treated with standard drug [MTX; 0.3 mg/kg BW]. Cells size was measured and by image analysis using the ImageJ software and affected in induced animals while this size recovers upon using 26 and 50 mg/kg *X. caffra* seeds extract compared to control and treatment by standard drug. II-Apoptosis in osteoclasts cultured from normal, induced rats, induced and treated with 26, 50 and 100 mg/kg *X. caffra* seeds extract, induced rats and treated with standard drug were detected by flow cytometry using Annexin V-FITC and propidium iodide on the cells. Where number of cells were affected in induced animals while this number recover upon using 26 mg/kg *X. caffra* compared to control and treatment by standard drug. (*order of flow cytometry results is same order like of microscopic osteoclast examinations).

To investigate the effect of *X. caffra* seeds extract on osteoclast structure, isolated osteoclasts from different experimental groups were cultured for seven days and examined using an inverted microscope. A significant decrease (*p* < 0.05) was observed in the size of osteoclast cells isolated from induced rats compared to cells from the negative control group. In contrast, osteoclasts cultured from animals treated with 26 mg/kg of *X. caffra* seeds extract regained their normal morphology, resembling that of the negative control group. A slight change in osteoclast size was observed in cultured cells from animals treated with 50 and 100 mg/kg of *X. caffra* seeds extract, as well as MTX-treated rats (Fig. 5I). These findings indicate that 26 mg/kg of *X. caffra* seeds extract represents an optimum dose for enhancing the maturation and preservation of osteoclast structure in treated arthritic rats. The apoptosis rate was measured ex *vivo* in osteoclasts using an Annexin V staining kit and flow cytometry. The apoptotic rate in the normal group was 19 ± 1.7%, whereas it significantly increased to 44.8 ± 4.6% in cultured cells from induced animals (*p* < 0.05), indicating an elevated rate of cell death. Treatment with 26 mg/kg of *X. caffra* seeds extract was found to be optimal in restoring osteoclast morphology and normalizing apoptosis levels, comparable to the standard drug-treated group (Fig. 5II). Similarly, Hong et al.^50^ reported that *Chrysanthemum zawadskii* regulated osteoclast development and mitigated inflammatory colitis and arthritis in experimental animals. Consistent with their findings, our results demonstrated that 26 mg/kg of *X. caffra* seeds extract played a notable role in modulating osteoclast apoptosis in induced arthritic rats.

### Effect of ***X. caffra*** extract on cytokines production

**Fig. 6:**
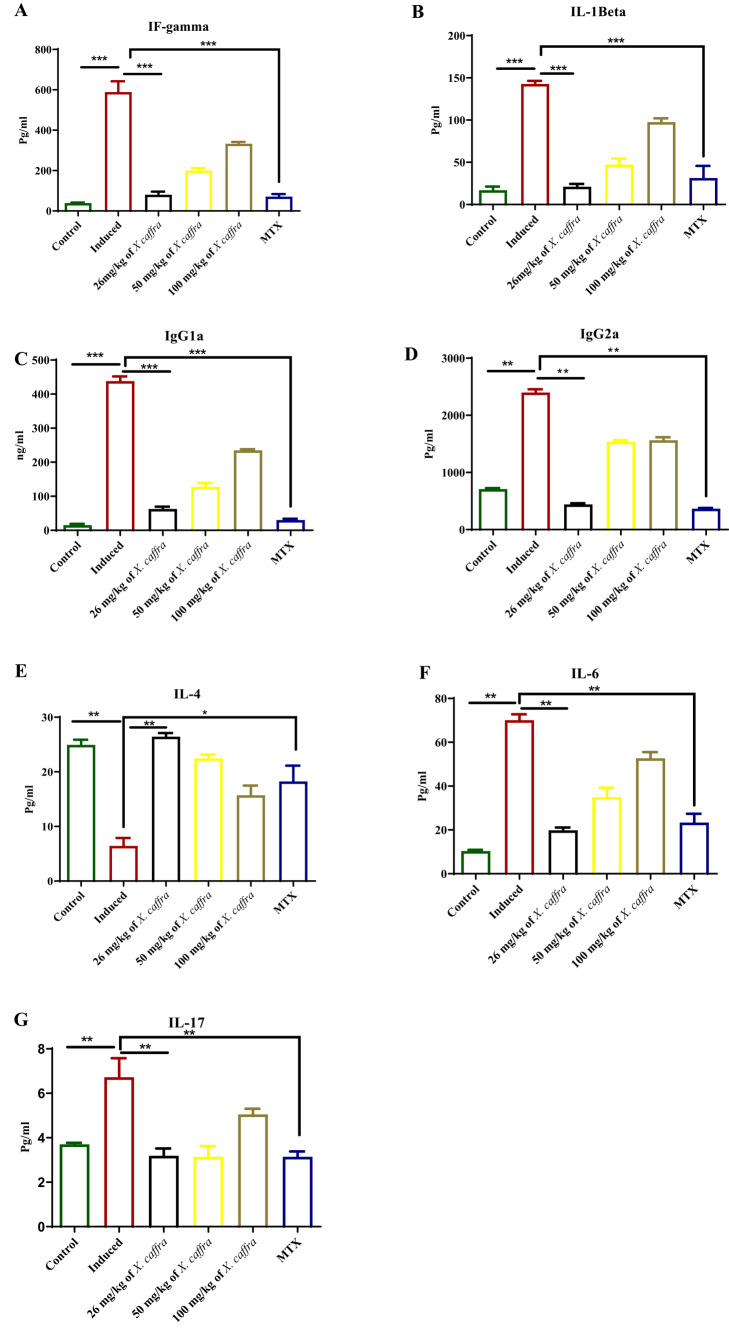
Effects of 26, 50 and 100 mg/kg *X. caffra* seeds extract on the levels of cytokines in serum of AIA rats versus normal rats and upon treatment using MTX. Pro-and anti-inflammatory cytokine levels in serum were measured using ELISA. (**A**) IFN-γ, (**B**) IL-1β, (**C**) IgG1a, (**D**) IgG2a, (**E**) IL-4, (**F**) IL-6 (**G**) IL-17. Values are presented as mean ± SEM of nine animals per group. **p < 0.05, ***p < 0.01.

Cytokine (IFN-γ, IL-1β, IgG1a, IgG2a, IL-4, IL-6, and IL-17) concentrations were measured at the end of the experiment. Serum samples were collected, and cytokine levels were determined using an ELISA kit. Cytokine levels differed between the control and treated groups. The results showed how 26, 50, and 100 mg/kg of *X. caffra* seeds extract affected cytokine expression in rats. In normal rats, the tested cytokines were within the normal range, and no inflammatory response was observed in the negative control group. In contrast, in the antigen-induced arthritis (AIA) group, the levels of IFN‐γ, IL-1β, IL-6, and IL-17 were significantly increased (*p* < 0.05), while IL-4 levels were significantly decreased (*p* < 0.05). Treatment with 26 mg/kg of *X. caffra* seeds extract exhibited a highly protective effect, as both pro- and anti-inflammatory cytokine levels returned to near-normal values, comparable to those observed in methotrexate-treated animals (0.3 mg/kg body weight). Treatment with 50 and 100 mg/kg of *X. caffra* seeds extract also produced a beneficial effect on cytokine levels in induced animals, though less pronounced than that observed with the 26 mg/kg dose (Fig. 6).The release of inflammatory mediators such as IL-1β, TNF-α, IL-6, IL-17, COX-2, and 5-LOX plays a key role in the progression of inflammation, alteration of joint function, synovial degeneration, cartilage destruction, and bone remodeling. IL-1β stimulates osteoclast activation, leading to bone damage, while IL-6 contributes to immune regulation and osteoclast differentiation. Moreover, IL-17 has a crucial role in rheumatoid arthritis (RA) by promoting excessive production of pro-inflammatory cytokines and matrix metalloproteinases (MMPs), as well as enhancing osteoclast activation^51,52^. The present study revealed that treatment with *X. caffra* seeds extract significantly reduced the concentrations of IFN-γ, IgG1a, IgG2a, IL-1β, IL-6, and IL-17, while enhancing IL-4 production, confirming the anti-arthritic activity of 26 mg/kg of *X. caffra* seeds extract through the downregulation of pro-inflammatory mediators and the upregulation of anti-inflammatory cytokines in the experimental rat model.

### Effect of ***X. caffra*** extract on liver and kidney functions

**Fig. 7:**
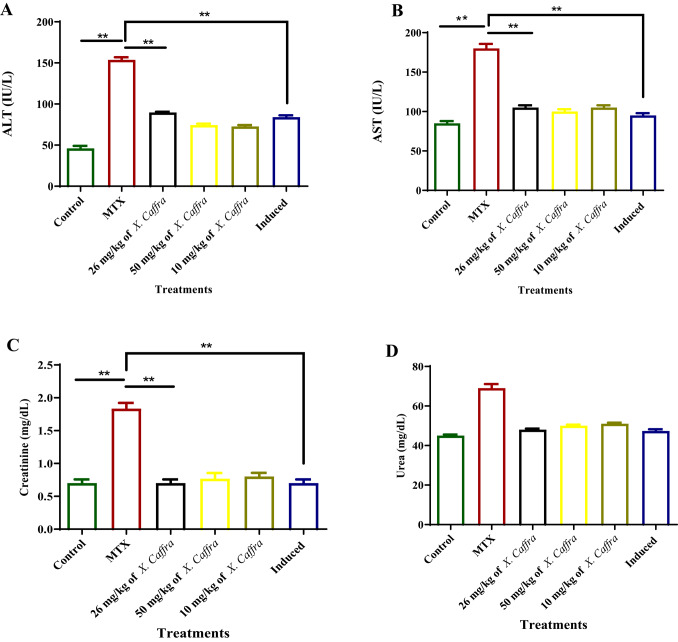
Effects of 26, 50 and 100 mg/kg *X. caffra* seeds extract on liver and kidney functions levels in serum of tested animal groups. The values were measured using biochemical Kits. (**A**) ALT, (**B**) AST, (**C**) Creatinine, (**D**) Urea, Values are presented as mean ± SEM of nine animals per group. **p < 0.05.

To test the effect of a mixture of active components from *X. caffra* seeds extract on liver and kidney functions in all tested rat groups in this study, biochemical tests were conducted. ALT and AST were measured as markers of liver function (Fig. 7A and B). ALT and AST values were significantly increased in the MTX group (*p* < 0.05) compared to the control group, while only slight increases were observed in the induced and treated animals receiving different doses of *X. caffra* seeds extract. Creatinine and urea were evaluated as markers of kidney functions (Fig. 7C and D). It was observed that creatinine levels were significantly elevated (*p* < 0.05) in the MTX group, accompanied by a slight increase in urea levels in the same group. However, the other tested animal groups exhibited kidney function values comparable to those of the control group, indicating the safety of *X. caffra* seed extract on hepatic and renal functions in the tested animals. MTX is commonly used in low doses to treat rheumatoid arthritis (RA) patients, but its use is limited due to its harmful effects on various tissues and organs resulting from its strong oxidative activity^53,54^. In the present study, *X. caffra* seeds extract demonstrated minimal effects on liver and kidney functions, suggesting that it can be safely used in the treatment of MTX-induced toxicity in rats.

## Conclusion

The results of this study showed that *X. caffra* seeds aqueous ethanolic extract has strong anti-inflammatory and anti-arthritic properties in both in vitro and in vivo settings. Administration of 26 mg/kg of the extract had maximally reduced the symptoms of arthritis, improved protective mediators (IL-4), decreased inflammatory cytokines (IL-1β, IL-6, IL-17, and IFN-γ), and restored joint structure. Bioactive polyphenols and flavonoids, especially those with established immunomodulatory and antioxidant properties, are probably responsible for these biological effects. Besides, the results imply that *X. caffra* seeds extract may provide a safe and efficient natural alternative for the treatment of rheumatoid arthritis with low hepatic and renal toxicity. It should be noted that this study has several limitations including: (1) The present results are based on an animal model, and it is yet unknown if they apply to rheumatoid arthritis in humans. (2) Chronic toxicity and pharmacokinetics were not thoroughly examined; only one type of extract and a small dose range were assessed. (3) The exact chemical and molecular processes behind the observed immunomodulatory and anti-inflammatory actions were not entirely understood. (4) Reproducibility may also be impacted by variations in phytochemical composting upon change of ecological conditions surrounding the plants. Thus, more research involving a variety of extracts, longer treatment periods, mechanistic evaluations, and clinical trials is required to confirm *X. caffra*’s therapeutic potential for rheumatoid arthritis.

## Supplementary Information

Below is the link to the electronic supplementary material.

Supplementary Material 1

## Data Availability

The datasets used and/or analyzed during the current study available from the corresponding author on reasonable request.

## Abbreviations

LC-HRMS: liquid chromatography high-resolution mass spectrometry
LOX enzyme: lipoxygenase enzyme
CFA: Complete Freund’s Adjuvant
MTX: Methotrexate
