# Supplementary material for: In vitro anti-inflammatory potential and in vivo anti-arthritis activities of Ximenia caffra extract on antigen-induced arthritis in rats
Source: Sci Rep. 2026 Jan 6;16:797. doi: 10.1038/s41598-025-32300-7 (PMC12779950; doi:10.1038/s41598-025-32300-7)
Supplement: Supplementary file 1 — Supplementary Material 1 [file 41598_2025_32300_MOESM1_ESM.docx]

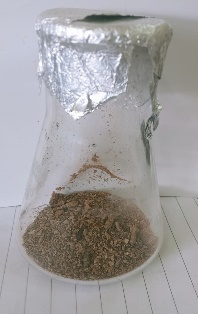


**(Supplematary Figure 1)** *X. caffra* grounded seeds for extraction.


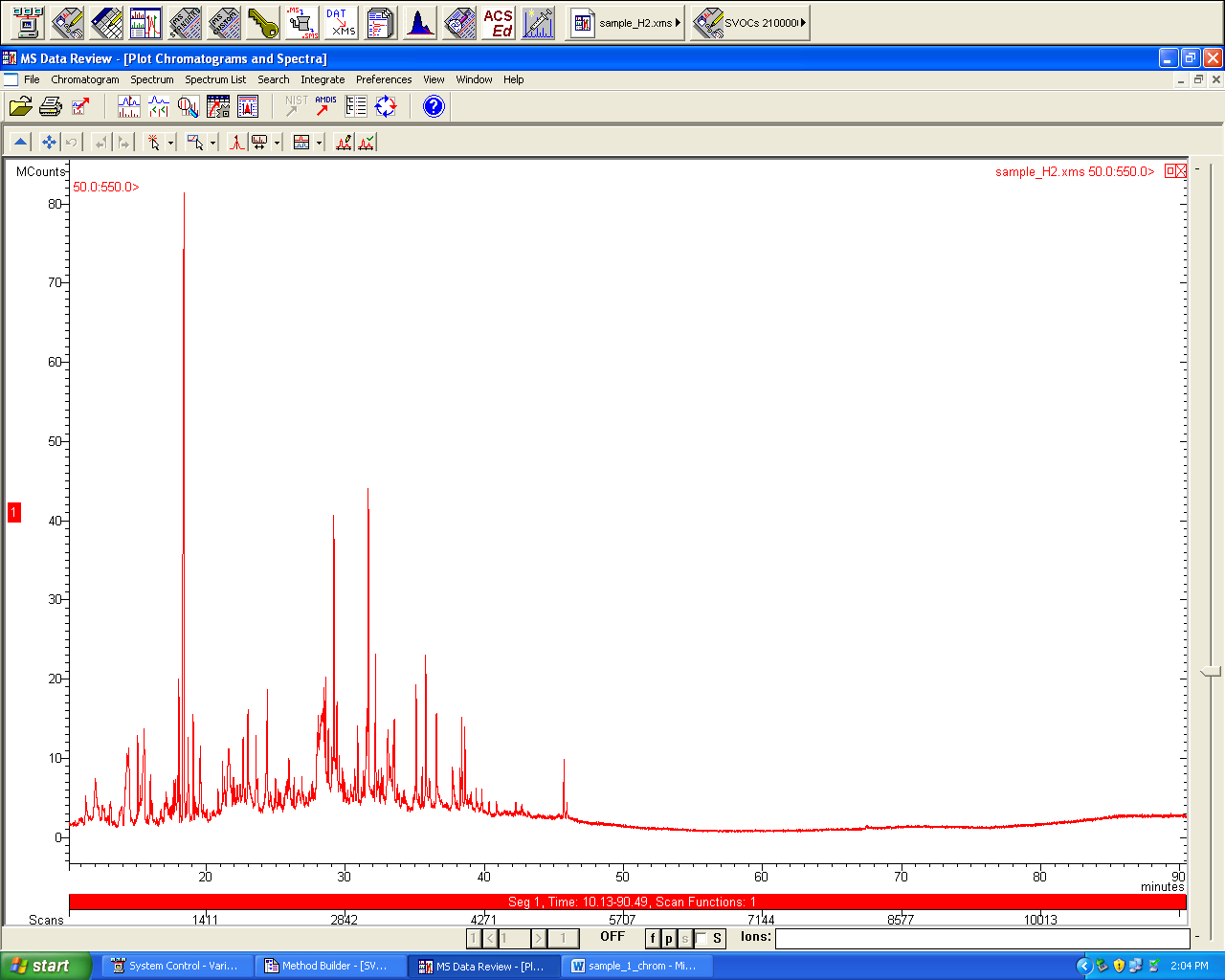


(**Supplematary Figure 2**) LC-HRMS Chromatogram for compounds obtained for from *X. caffra* extract.
